# Phenotypic plasticity in plasmodial slime molds and molecular phylogeny of terrestrial vs. aquatic species

**DOI:** 10.1007/s12064-022-00375-9

**Published:** 2022-08-27

**Authors:** T. Hoppe, U. Kutschera

**Affiliations:** 1 Research Group for Biology Education, Institute for Zoology and Evolutionary Research, Faculty of Biological Sciences, Friedrich-Schiller-University Jena, Am Steiger 3, 07743 Jena, Germany; 2 AK Evolutionsbiologie, Neuburg, 79104 Freiburg i. Br., Germany

**Keywords:** Aquatic myxomycetes, Didymiaceae, Ecology, Phenotypic plasticity, Phylogeny, 18S-rDNA

## Abstract

Fifty years ago, the enigmatic Brazilian myxomycete-species *Didymium aquatile* was described and analyzed with respect to the structure of the plasmodium and its spores. In this study, we compare this rare plasmodial slime mold with another, temporarily aquatic taxon from Europe, *Didymium nigripes*. Phenotypic plasticity of *D. nigripes* was investigated under various environmental conditions. Large changes in the morphology of the plasmodia were observed. For species identification, characteristics of the fruiting bodies are key features. However, *Didymium aquatile* was only characterized by its “abnormal” plasmodia, but no molecular data were available. Here, we analyzed DNA-sequences of 22 species of the genera *Didymium* and *Diderma* with a focus on this South American taxon via molecular genetics. A comparison of 18S-rDNA-sequences from *D.* *aquatile* and 21 other *Didymium* (and *Diderma*)-species indicates that *D.* *aquatile* is a reproductively isolated morpho-species. Phenotypic plasticity of *D. nigripes* is documented with respect to plasmodium morphology and the formation of fruiting bodies, as an example of an adaptation of a terrestrial species to aquatic environments.

## Introduction

Plasmodial slime molds (myxomycetes) are a group of terrestrial protozoans comprised of ca. 1000 described species (Stephenson and Rojas 2017; Baba and Sevindik 2018). The haploid myxamoebae feed on bacteria or other microorganism, whereas the multinucleate plasmodium grows on various organic substrates (fungal hyphae, algae, bacteria, etc.). After an internal or environmental stimulus, the so-called plasma-organism transforms into a fruiting body (Hoppe and Kutschera 2015; Kutschera and Hoppe 2019).

The myxomycetes can be divided into five (formerly 6) orders (Hoppe and Kutschera 2010; Fiore-Donno et al. 2007; Stephenson and Rojas 2017), wherein the Physarales represent one of the most diverse taxa of these groups. In this order, members of the genus *Didymium* are divided into approx. 89 morpho-species (Lado 2001; Bellido et al. 2017).

Myxomycetes have no cell wall. This makes them very motile, and the thin membrane predisposes them to be found frequently in high humidity habitats. Most myxomycetes live and reproduce on land, but there are also reports on species adapted to in aquatic environment (especially within the order *Physarales*; Ward 1886; Gray and Lanning 1978; Kappel and Anken 1992; Lindley et al. 2007; Stephenson and Rojas 2017).

Fifty years ago, a species was described as being a myxomycete entirely adapted to an aquatic habitat. Accordingly, Nannenga-Bremekamp and Gottsberger (1971) named this enigmatic species from São Paulo, Brazil (South America) *Didymium aquatile* (Nann.-Bremek. and Gottsb.). Due to its rarity, there is a scarcity of information about this enigmatic taxon.

The fruiting bodies of species of the genus *Didymium* are presented by plasmodiocarps or sporocarps. The thin peridium is covered with crystalline calcium carbonate. In contrast to the peridia, the capillitium is almost lime-less; a columella is usually present. In large quantities, spores are black, and by microscopic investigation, they appear to be brown (Martin and Alexopoulos 1969). These characteristics were also described for *D. aquatile* (Nannenga-Bremekamp and Gottsberger 1971).

The well-studied representatives of the Didymiaceae are heterothallic (mictic) and non-heterothallic (apomictic) species (Clark 2004). Reproduction in many myxomycetes shows a variety of possible strategies (Clark and Haskins 2013, 2014). In the majority of myxomycetes, the life cycle consists of an alternation between diploid (plasmodial) and haploid (myxamoebal) nucleoid phase (Feng et al. 2016, Kutschera and Hoppe 2019, Hoppe et al. 2014).

In contrast to genetic adaptation, morphological changes in response to an environmental stimulus are also observed for many myxomycetes (i.e., phenotypic plasticity, Clark 2004). These characteristics are maintained over a specific period of time, and may be problematic for the exact determination of species status, particularly if the alterations are found in the fruiting bodies.

Thirteen years ago, samples of the species *D. nigripes* were isolated from an aquarium to study the plasticity of plasmodia and fruiting body characteristics (Müller et al. 2008). This rare myxomycete displays a striking plasmodium, which is similar to that of Gottsberger and Nannenga-Bremekamps’ drawings of 1971. It is possible that the enigmatic Brazilian species *D.* *aquatile* is an ecological variant of a better-characterized member of the genus *Didymium*. Therefore, we studied the morphology, phenotypic plasticity, and molecular systematics of 24 species of myxomycetes with the aim of finding out whether or not *D. aquatile* is in fact a separate morpho-species.

## Materials and methods

A plasmodium of an aquatic myxomycete was isolated from a freshwater aquarium (Müller et al. 2008). The plasmodium (strain MYX51) was divided and cultivated in aquatic milieu and on water-agar (6–8 g agar/ 1 L ddH_2_O) at 25 °C (darkness). Oat (*Avena sativa*) meals were sterilized and used as carbohydrate-rich food source.

In both cultivated strains, the sporocarps developed after about three months. Scanning Electron Micrographs (SEM-Images) were obtained as described by Hoppe and Kutschera (2010) for *D.* *nigripes* MYX51 (aquatic and terrestrial line, Herbarium of the Natural History Museum Gera, Germany) and *D. aquatile* MYX463 (= Gottsberger 11–7820, Herbarium of the University of Ulm, Germany).

The spore ornamentations for MYX51 and MYX463 were detected with the program “ImageJ” for SEM-micrographs. A measuring range of *d* = 2.41 microns was created for automated detection. In addition, the incision in several spores was morphologically characterized.

Genomic DNA was isolated from spores of these two strains, and 20 other *Didymium* and *Diderma* species, which represent the second largest group of species within the genus *Didymium* (Table 1). The isolation protocol for DNA-extractions as described by Hoppe (2013) was used.

**Table 1:** Species investigated, inclusive collection data and NCBI-Accession numbers (KU577264 is a new sequence)

Species	Herbar number	Locality	Accession number
*Didymium melanospermum*	HoppeMYX488	Oberholzklau, Germany	KU577268
*Didymium melanospermum*	HoppeMYX526	Obersetzen, Germany	KU577269
*Didymium melanosporum*	HoppeMYX333	Riedau/Habach, Austria	KU577267
*Didymium melanospermum*	HoppeMYX507	Hilchenbach, Germany	KU577270
*Didymium ochroideum*	HoppeMYX297	Bad Ischl/Kaltenbach, Austria	KM977860
*Didymium nivicolum*	HoppeMYX308/Nowotny5552	Tarsdorf/Filzmoos, Austria	KP323376
*Didymium verrucosporum*	HoppeMYX226/Mueller 1892	Madeira, Spain	KP323377
*Didymium comatum*	HoppeMYX185/Mueller 2246	Thuringia, Germany	KP323374
*Didymium dubium*	HoppeMYX231/Mueller 2016	Thuringia, Germany	KP323375
*Didymium balearicum*	HoppeMYX230/Mueller 2287	La Palma, Spain	KP323373
*Didymium flexuosum*	HoppeMYX295/Nowotny5898	St. Margareten/Rosental, Austria	KM977857
*Didymium iridis*	HoppeMYX50/Mueller 2689	Zypern, Greek	KU577266
*Didymium nigripes*	HoppeMYX51	Kassel, Germany	KM977859
*Didymium crustaceum*	HoppeMYX235/Mueller 2543	Thuringia, Germany	KM977856
*Didymium aquatile*	HoppeMYX463/Gottsberger11-7820	Rubiao Junior/Botucatu, Brazil	KU577264
*Didymium bahiense*	HoppeMYX214/Mueller 1486	Thuringia, Germany	KP323372
*Didymium minus*	HoppeMYX75	Ebeleben, Germany	KM977858
*Didymium minus*	HoppeMYX496	Oberholzklau, Germany	KU577271
*Diderma deplanatum*	HoppeMYX440/Nowotny 9245	Niederranna/Rannatal, Austria	KM977850
*Diderma hemispaericum*	HoppeMYX436/Nowotny 7402	Bad Ischl/Kaltenbach, Austria	KM977853
*Diderma chondrioderma*	HoppeMYX439/Nowotny 8952	Grieskirchen, Austria	KM977850
*Diderma globosum* var. *europaeum*	HoppeMYX443/Nowotny 13192	Bad Ischl/Hoisnrad-Alm, Austria	KM977852
*Diderma niveum*	HoppeMYX442/Nowotny 10817	Strobl/Postalm, Austria	KM977854
*Diderma radiatum*	HoppeMYX437/Nowotny 8198	St. Paul/Isere, France	KP323371

A partial sequence of the 18S-rDNA was amplified using a PCR-protocol as detailed by Hoppe (2013). The following myxomycete-specific primers were used: S3b (Hoppe and Schnittler 2015) and S31R (Hoppe 2017). The PCR-products were analyzed via Beckman-Coulter Diagnostics (Krefeld, Germany). The sequences were analyzed and visualized by MEGA 6.06 (Table 1). A phylogenetic tree was calculated with Neighbor-Joining methods, based on sequences of 237-bp length, including an intron and a highly conserved region of the genome.

## Results

### Morphological studies

**Fig. 1:**
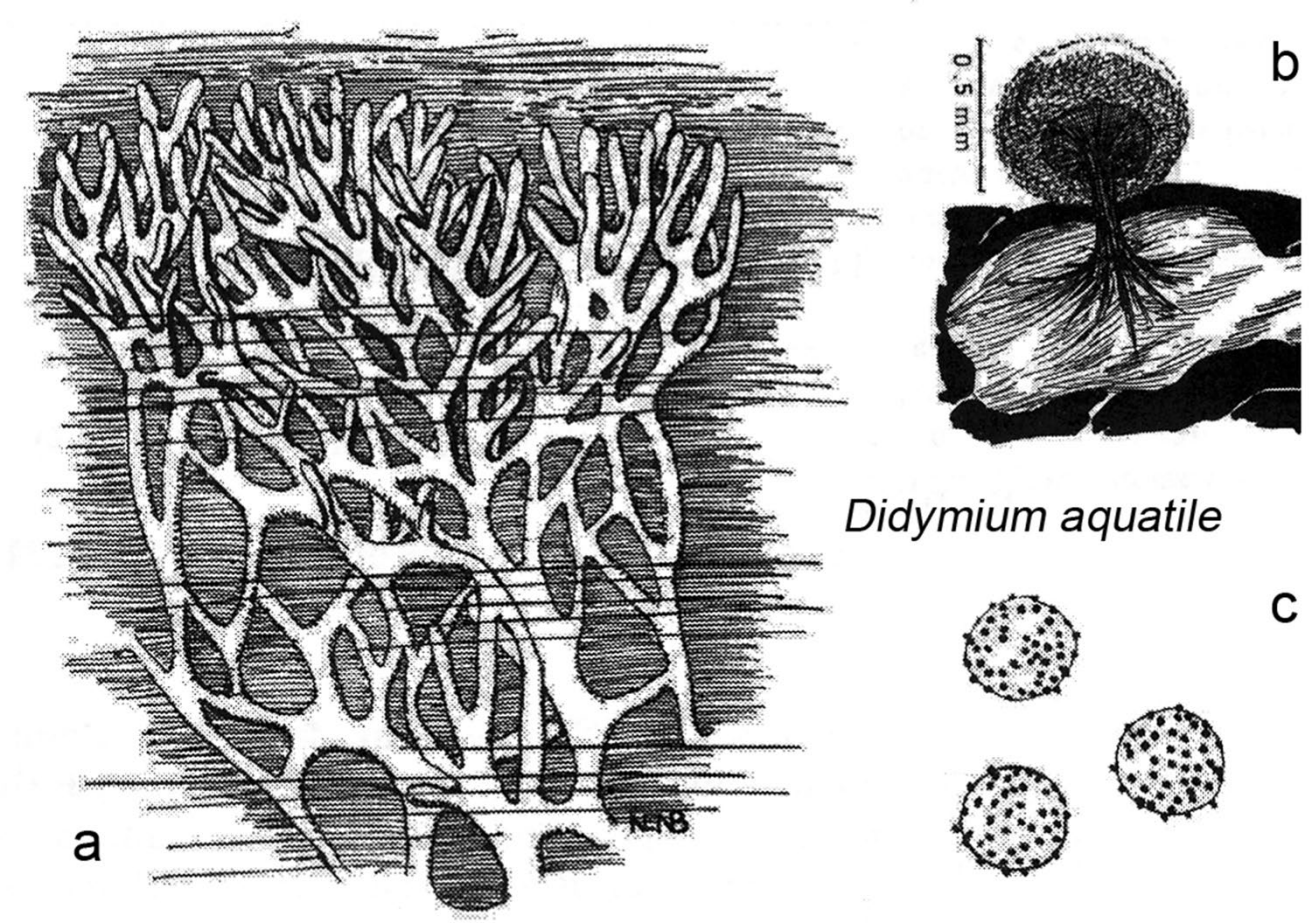
Morphology of the plasmodium (**a**), sporangium (**b**) and spores (**c**) of the South American myxomycete *Didymium aquatile* MYX463 (Gottsb. and Nann.-Bremek.), reproduced from the original description of this enigmatic species (adapted from Nannenga-Bremekamp and Gottsberger 1971)

**Fig. 2:**
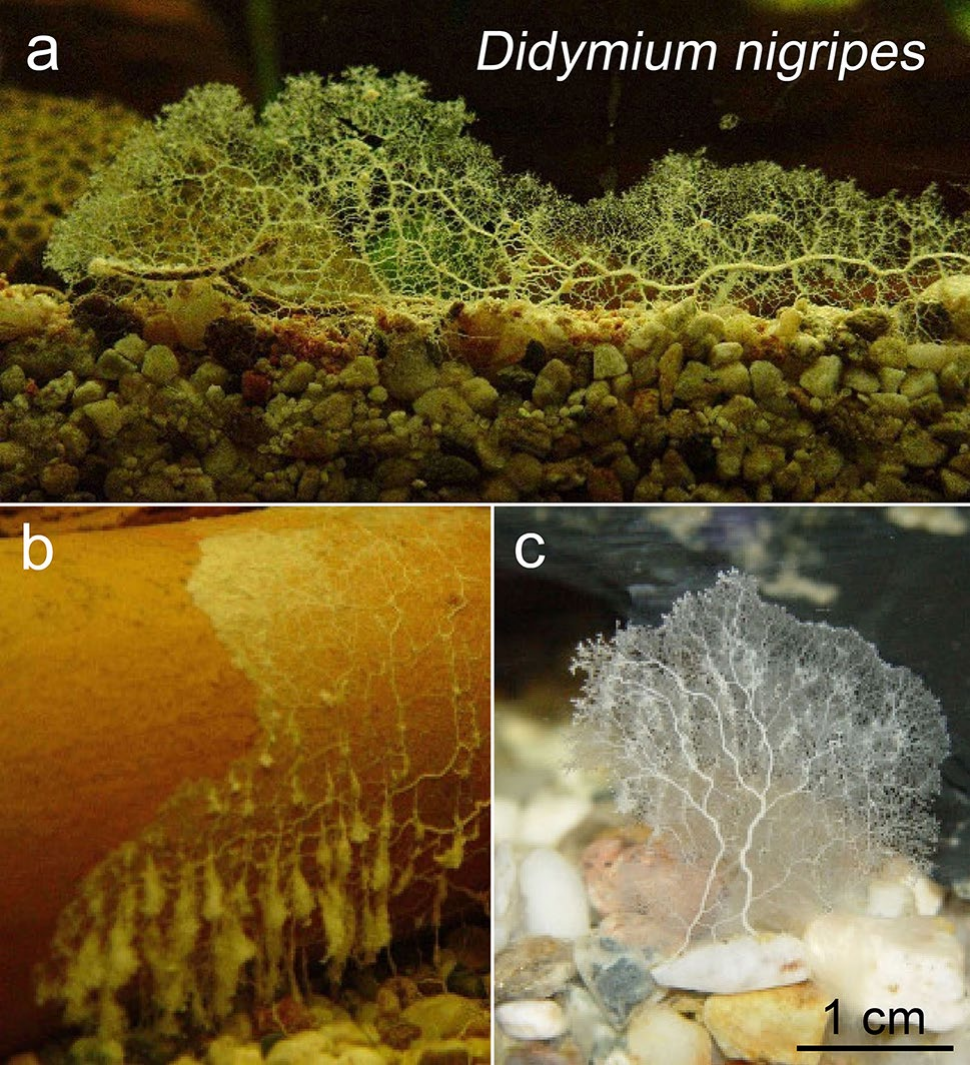
Morphology of the plasmodia of the semi-aquatic myxomycete *Didymium nigripes* (MYX51), grown in a freshwater aquarium. Three different individuals of the same species are depicted (**a**, **b**, **c**) (adapted from Müller et al. 2008)

In 1997, the Brazilian myxomycete *Didymium aquatile* (Nann.-Bremek. and Gottsb.) was described as an aquatic morpho-species. Figure 1 shows a reproduction of Nannenga-Bremekamp and Gottsberger’s fifty-year-old drawings. The plasmodium (Fig. 1a) and sporocarp/spores (Fig. 1b, c) are characterized by species-specific features. We performed a detailed morphological investigation of the plasmodium of *Didymium nigripes* (Fig. 2). These results indicate that *D.* *aquatile* from South America and *D. nigripes* from Europe represent morphologically similar taxa.

### Molecular phylogenetic tree based on rDNA-sequences

**Fig. 3:**
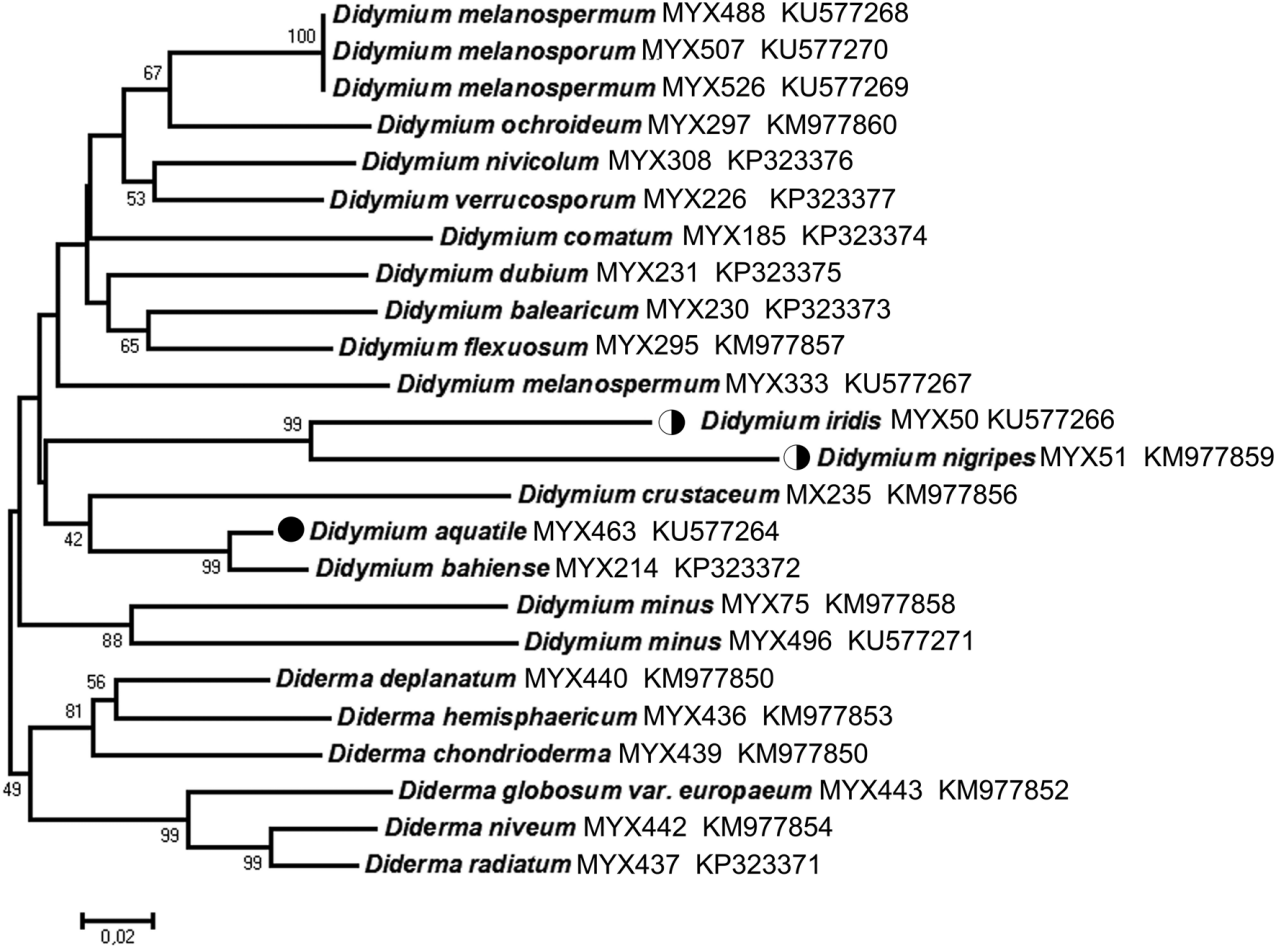
Phylogenetic tree reconstructed from 18S-rDNA-sequences, using the Neighbor-Joining method (100,000 replicates in the bootstrap test). The evolutionary distances were computed using the Tamura 3-parameter method and are in the units of the number of base substitutions per site. Codon positions included were 1st + 2nd + 3rd + Noncoding. All positions containing gaps and missing data with less than 98% site coverage were eliminated. There were a total of 237 positions in the final dataset

No genetic material has yet been extracted from the South American taxon shown in Fig. 1, and, accordingly, no DNA-sequence-based tree that includes this species has yet been constructed. Therefore, we investigated 22 species of myxomycetes (17 representatives of the genus *Didymium* and 5 of the genus *Diderma*), using the tools of molecular genetics. A 375 bp-sequence from the 18S-rDNA was obtained from samples of the Brazilian *Didymium aquatile*, and a 237-bp fragment was used for phylogenetic investigations. PCR-amplifications for longer fragments failed, probably due to strong degeneration of the stored genomic material. Phylogenetic trees were calculated for these Didymiaceae, using Neighbor-Joining methods. One representative evolutionary scheme is depicted in Fig. 3. Our isolated aquatic strain (Fig. 2) MYX51 matched genetically with *D. nigripes* (Link) Fr., indicating that these taxa represent identical morpho-species. Our phylogenetic tree further documents that (1) the three different isolates of *Didymium melanospermum* are genetically identical; (2) all 16 morpho-species of the genus *Didymium* form a monophyletic group; (3) the two semi-aquatic myxomycetes *D. iridis* and *D. nigripes* are sister-taxa; (4) *Didymium aquatile* from South America is most closely related to *D. bahiense* from Thuringia, and (5) all 6 morpho-species of the genus *Diderma* form a cluster. These results document that *D. aquatile* is a separate species and not a variant of other semi-aquatic myxomycetes.

### Spore morphology and anatomical studies

The morphology of the plasmodia of MYX51 (*D. nigripes*) was investigated for different environmental conditions.

In aqueous milieu, a typical phaneroplasmodium was formed. Many ends of the veins were present infiltrating in the aqueous milieu in (Fig. 5a). Immediately after transferring on water-agar, MYX51 produced exo-enzymes and liquefied its substrate (to a larger extent than other myxomycete-plasmodia in the laboratory). This increased expression ceased after a few weeks. The typical plasmodial morphology of a myxomycete was observed depending on the water content of the agar.

**Fig. 4:**
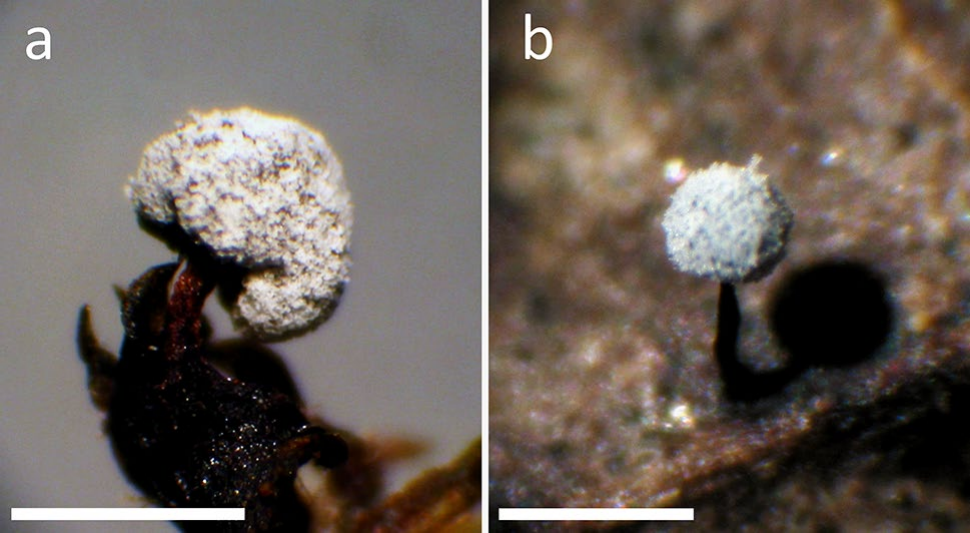
Photographs of sporocarps from *Didymium aquatile* (MYX463) (**a**) and *Didymium nigripes* (MYX51), raised in terrestrial environment (**b**); Scale bars are 1 mm

**Fig. 5:**
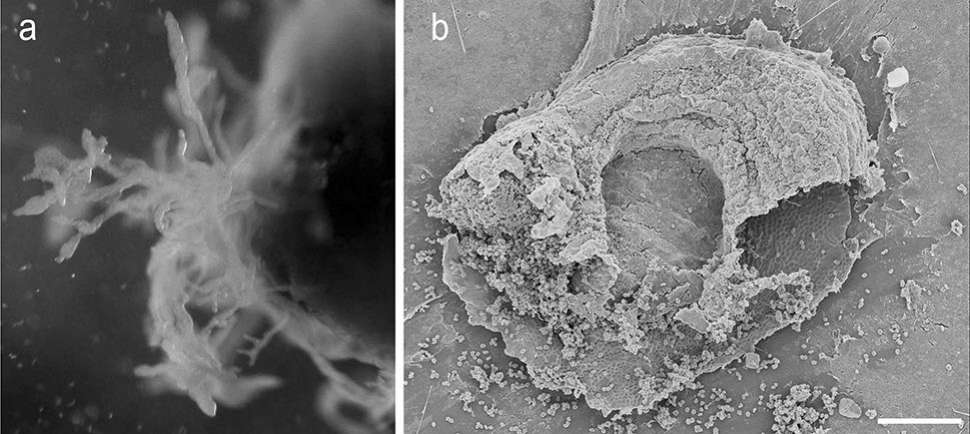
Microscopic micrographs of *Didymium nigripes* MYX51 Plasmodium raised under aquatic conditions (**a**), and a typical sclerotium, in aquatic environment (**b**); Scale bars are 0.5 mm

Varying vein formations were observed. Under terrestrial and aquatic conditions, fruiting bodies were formed. The terrestrial strain formed sporocarps (Figs. 4a, b, 5b).

**Fig. 6:**
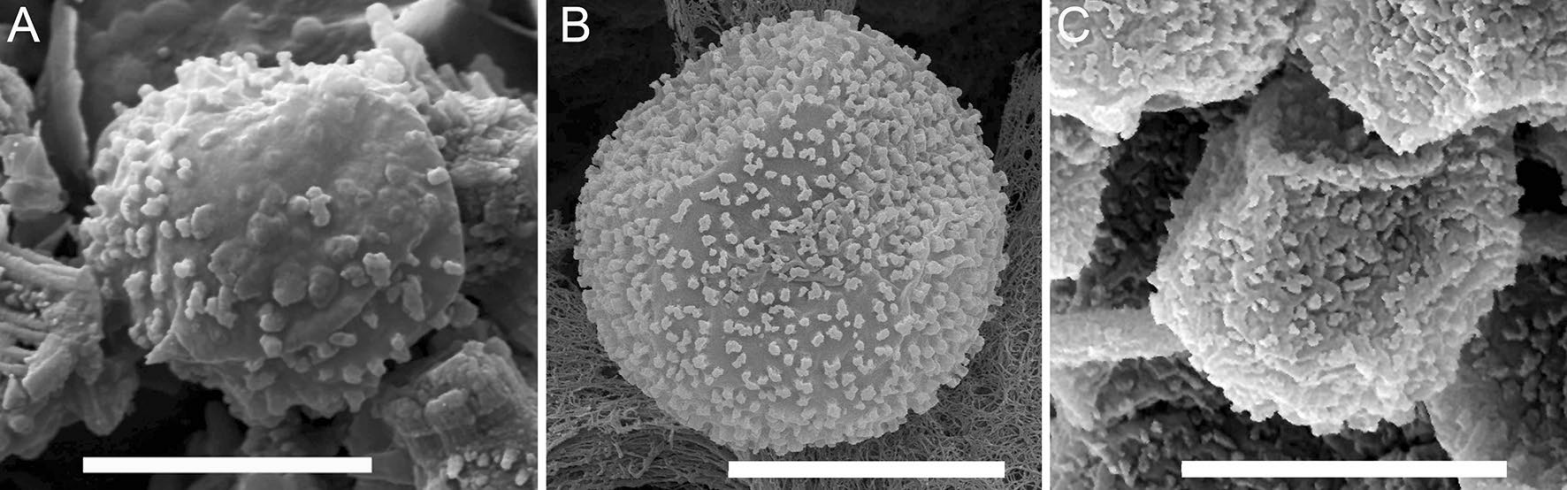
Scanning electron micrographs of spores from *Didymium aquatile* (MYX463) (**a**) and *Didymium nigripes* (MYX51), (**b**) terrestrial environment. In addition, a spore of *Didymium nigripes* (MYX51) is shown, raised in aquatic environment (**c**); Scale bars are 5 µm

The fruiting bodies and spores of MYX51 (terrestrial line) were similar to those described in the literature (Figs. 4, 6). In contrast, under aquatic conditions, non-calcareous plasmodiocarps were formed. Capillitial were almost completely missing. The spores were covered with coarse spines (Fig. 6b, c).

The fruiting bodies of *D. aquatile* were described by Gottsberger and Nannenga-Bremekamp (1971). Spores were examined, using scanning electron microscopy; the propagules had conspicuous rough spines (Fig. 6a).

In terrestrial and aquatic strains of *D. nigripes*, 15.3 spikes versus 14.8 spikes (Fig. 6b, c) were counted. Therefore, the number of spines per spore within the measuring circle was very similar. However, the spines of *D. nigripes* (aquatic) appear more prominent. For the spores of *D. aquatile*, 12 spikes were counted per measuring range.

## Discussion

To determine the taxonomic status of myxomycetes, subtle distinctions in morphology have been analyzed and described, and fruiting bodies are considered to be the most relevant (Schnittler and Mitchell 2000). The high phenotypic plasticity of these organisms with respect to the interaction of the plasmodium with its environmental conditions may lead to variable features of the organic structure as a whole.

The morphological changes are not necessarily hereditary, but rather may be a form of adaptive plasticity. Nevertheless, it allows for increased tolerance of special environmental conditions and increases the fitness of an organism in the corresponding ecosystem (Ghalambor et al. 2007). As a result, these genotypes are selected, which best adapt to environmental changes (Winsett and Stephenson 2013). In apomictic, clonal lines, which are frequently found in other *Physarales*, these genotypes can quickly adapt and dominate the evolving population (Alexopoulos 1960).

Several scientists have pointed out that these problems are unresolved and formulated criteria for species descriptions (Schnittler and Mitchell 2000; Lado 2001). Although these criteria are desirable, they are difficult to enforce in this group of organisms. Even the collection of rare species leads to difficulties. Relatively few myxomycetes can be cultivated under laboratory conditions and even develop fruiting bodies in a reproducible way. Analysis of suitable genes may provide a workable solution.

We used the *D. nigripes* strains MYX51 and *D. aquatile* to explore the taxonomic status of these organisms. The species delimitation took place mainly on the basis of a rare microhabitat for myxomycetes. Moreover, the shape of the plasmodium was added to the characteristics of the fruiting body. In this case, the organism was examined under laboratory conditions for a longer time under different environments.

While the features of the unusual habitat for a myxomycete plasmodium and fruiting body initially made the appearance to be an aquatic variation, the species was identified as *D. nigripes* after sequencing and comparing this to known sequences. After changing the microhabitat characteristics, the plasmodial morphology resembled the already described plasmodia of *D.* *nigripes*. It was possible to study the plasticity of various characteristics in the life cycle of the strain MYX51. However, the ornamentation of the spores and the genetic constitution remained constant. Through a genetic comparison with herbarium material of *D. aquatile* and other *Didymiaceae*, we have shown that this species, that has been ignored over the past four decades, can be genetically separated from the aquatic strain MYX51, and morphologically from other similar taxa.

Genetic adaptation results in a population with a particular average phenotype (Fischer 1958). However, within an interbreeding group of organisms, genotypically different individuals may exist. These have the capability for a specific range of variation (i.e., phenotypic plasticity, Pigliucci et al. 2006). This malleability of the phenotype can interact with changing environmental conditions so that an adapted organism has a higher reproductive success.

Many myxomycetes have spread over large areas and habitats. So far, no exact information about the real distribution of different species is available. Nevertheless, local marginal areas are also available within a distribution range of a species. These are habitats that may have favorable minimum conditions (extreme drought, water, low temperature). This study documents that further investigations into the habitats of these amoebae-like microbes are necessary to get a more complete knowledge of niche occupation. Under moist-chamber conditions, the cultivable myxomycetes fructify. Thus, the two features, i.e., morphology and genetics, should be studied in more detail (see Kamono et al. 2013; Feest 1987; Dahl et al. 2017; Therrien et al. 1977; Stephenson et al. 2008).

It is possible that the colonization of an aquatic habitat for most terrestrial species of myxomycetes is merely an opportunity to cope with problematic environmental conditions. *D.* *nigripes* MYX51 and *D.* *aquatile* were both able to be cultivated outside of their natural aquatic milieu. In terrestrial habitats, they form typical plasmodia with fruiting bodies (Nannenga-Bremekamp and Gottsberger 1971; Müller et al. 2008). The fruiting bodies of all species studied developed in a terrestrial milieu (sporocarps and spores). Although, in this group of organisms, the morphology during the life cycle is extremely variable, the ornamentation of the spores appears to be relatively constant. This feature is independent of phenotypic plasticity of plasmodia, or fruiting bodies.
